# Automated Capture and Analysis of Circulating Tumor Cells in Pediatric, Adolescent and Young Adult Patients with Central Nervous System Tumors

**DOI:** 10.3390/cancers15153853

**Published:** 2023-07-29

**Authors:** Wafik Zaky, Dristhi Ragoonanan, Izhar Batth, Long Dao, Jian Wang, Xueqing Xia, Najat C. Daw, Jonathan B. Gill, Soumen Khatua, Shulin Li

**Affiliations:** 1 Department of Pediatrics, The University of Texas MD Anderson Cancer Center, Houston, TX 77023, USA; 2 Department of Biostatistics, The University of Texas MD Anderson Cancer Center, Houston, TX 77030, USA

**Keywords:** liquid biopsy, circulating tumor cell, cell surface vimentin, pediatric, central nervous system tumors, diffuse midline glioma, H3K27M mutation

## Abstract

**Simple Summary:**

Central nervous system (CNS) tumors are the most common solid malignancy in pediatrics and prognosis remains poor for patients with relapsed or refractory disease. Currently, for most of these tumors, there are no biomarkers available to monitor treatment response or detect early recurrent disease. Patients are therefore subjected to serial imaging which provides limited information. There is an urgent need to develop minimally invasive, easily accessible biomarkers that can be used to monitor disease and help guide future treatment. Circulating tumor cells (CTCs) are cells that are shed from the primary tumor and can be detected from peripheral blood. In this first pediatric study, we show that the detection of CTCs is feasible and highly sensitive in patients with CNS tumors. Large-scale studies using our novel method of CTC detection will further help validate these findings and encourage large-scale implementation in future clinical trials.

**Abstract:**

Tumors of the central nervous system (CNS) are the most common and lethal childhood malignancy. Detection of residual disease and longitudinal monitoring of treatment response in patients are challenging and rely on serial imaging. This current standard of care fails to detect microscopic disease or provide molecular characteristics of residual tumors. As such, there is dire need for minimally invasive liquid biopsy techniques. We have previously shown the high specificity of using cell surface vimentin (CSV) to identify circulating tumor cells (CTCs) from patients bearing various types of cancers. Here, we describe the first report of CTCs captured from peripheral blood samples in 58 pediatric CNS tumor patients. In this study, we used a CSV-coated cell capture chip, the Abnova CytoQuest automated CTC isolation system, to boost the CTC capture from pediatric patients with CNS tumors. We successfully isolated CTCs in six glioma patients using immunostaining of histone H3 lysine27-to-methionine (H3K27M) mutations which are highly expressed by this tumor. We show that CSV is a viable marker for CNS CTC isolation and that this is a feasible method for detecting microscopic disease. Larger-scale studies focusing on CTCs in pediatric CNS tumors to explore their diagnostic and prognostic value are warranted.

## 1. Introduction

Central nervous system (CNS) tumors are the second most common malignancy in pediatrics, accounting for one out of four childhood cancer cases. Despite advances in treatment strategies, CNS tumors remain the leading cause of mortality in children with cancer [1,2]. Diagnosis relies mainly upon tissue biopsy which may be challenging depending on its anatomical location and has a high risk of morbidity. Current treatment approaches to CNS tumors include surgical resection, often limited due to their location and infiltrative nature, conventional chemotherapy, radiation and emerging targeted therapies. During and post-therapy, serial surveillance neuroimaging is the primary method for disease monitoring [3]. Reliance on repeated magnetic resonance imaging (MRI), the standard of care neuroimaging modality, however, poses several limitations, including: difficulty in differentiating pseudoprogression from true progression, inability to detect microscopic disease leading to potential delays in diagnosing relapsed disease, the need for sedation in young children given prolonged scanning times and lack of insight into the molecular characteristics of the tumor which may potentially guide targeted therapy [4,5]. Given these challenges and the sensitive neuroanatomical location of CNS tumors, there is a dire need to develop minimally invasive, novel approaches of detecting microscopic disease in patients with CNS tumors.

In the past few decades, there has been considerable effort in identifying a reliable, tumor-specific biomarker for patients with solid tumors. Peripheral blood-based biomarkers that have been studied in CNS tumors include extracellular vesicles, circulating tumor deoxyribonucleic acid and ribonucleic acid (ctDNA/ctRNA) materials and circulating tumor cells (CTCs) [6,7,8,9]. These CTCs are tumor cells that shed from the primary tumor and extravasate into the peripheral circulation and have repeatedly been reported as a reliable prognostic marker [10,11,12,13,14,15,16,17,18]. CTCs, however, are rare with only 1 cell per 10^6^–10^8^ peripheral blood mononuclear cells (PBMCs). The detection of CTCs has been focused on using size-based exclusion techniques or is dependent on molecular markers, the most commonly used being epithelial cell adhesion molecule (EpCAM). As a result, the primary focus of CTC-related studies has been epithelial tissue-based tumors [19,20,21,22,23]. However, the use of EpCAM for CTC detection limits its utility in patients with tumors that are EpCAM negative, have low expression, such as neurogenic tumors, or have mesenchymally transformed CTCs [18,24,25].

To address these deficiencies, our lab has developed a universal biomarker cell surface vimentin (CSV)-based CTC detection approach which can capture CTCs from both epithelial and mesenchymal cancer patients [26,27,28]. Using this method, we have demonstrated a highly sensitive and specific approach for capturing CTCs from the peripheral blood in these patients [26,28,29,30]. Despite the mounting evidence showing the clinical relevance of CTC enumeration and molecular analysis, there are few studies exploring the utility of CTCs in CNS tumors. Additionally, there is a lack of reproducibility in the detection of CTCs in patients with CNS tumors with detection rates varying from 20–39% in glioblastoma multiforme (GBM) patients to 77% in adults with Grade II–IV gliomas [31,32,33].

Given the limited data, further research is needed to determine the utility of CTC detection in CNS cancers. Moreover, there are no studies evaluating its role in pediatric CNS tumor patients. Herein, we present the first ever analysis using a unique and highly sensitive method of CSV CTC detection in the peripheral blood of fifty-eight pediatric patients with CNS tumors.

## 2. Materials and Methods

### 2.1. Patients and Data

The study was approved by the Institutional Review Board (or Ethics Committee) of the University of Texas at MD Anderson Cancer Center (Protocol: PA13-0014, 2014) and in accordance with the Declaration of Helsinki. All eligible patients with a confirmed diagnosis of a CNS tumor who presented to the Pediatric and Adolescent and Young Adult (AYA) Service between January 2014 and January 2020 were presented with the opportunity to enroll in this study. Informed consent was obtained from all subjects involved in this study. Peripheral whole blood samples were collected from 58 patients diagnosed with a CNS tumor, delivered to the lab for processing within 2–4 h of blood draw and processed within 24 h of delivery to the lab.

CTC data were analyzed along with data extracted from the electronic medical record including demographics, primary diagnosis, disease status at time of sample collection, prior chemotherapy and radiation therapy and the presence of genetic mutations.

### 2.2. Automated CTC Capture

**Figure 1:**
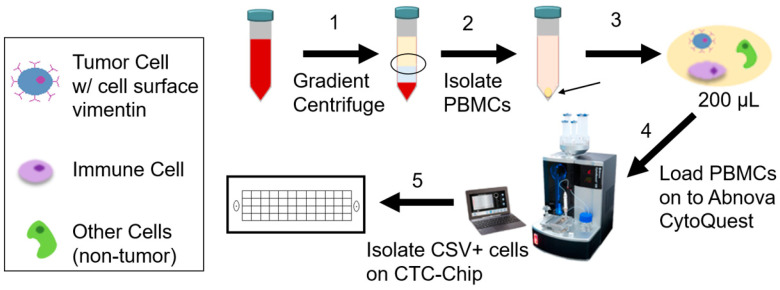
Circulating Tumor Cell (CTC) Isolation Workflow. Freshly collected (<24 h) patient peripheral blood is subjected to gradient centrifugation (1) to isolate peripheral blood mononuclear cells (PBMCs) (2). PBMCs are separated and collected into a 200 µL suspension (3) which is loaded onto the Abnova CytoQuest CTC isolation platform (4). After automated processing, CTCs are bound to the cell surface vimentin (CSV)-coated CTC chip (5). Cells captured on the chip are then stained (CSV, CD45, DAPI) and imaged.

As illustrated in Figure 1, freshly collected peripheral blood was diluted 1:1 with 2% fetal bovine serum (FBS) in phosphate-buffered saline (PBS) buffer. This was then loaded onto SepMate 50 (Stemcell Technologies, Vancouver, BC, Canada) containing 15 mL of Ficoll density gradient solution (Millipore Sigma, St. Louis, MO, USA). Following the manufacturer’s protocol, the solution was centrifuged at 1300× *g* force for 10 min at room temperature (RT). The separated peripheral blood mononuclear cell (PBMC) layer was poured into a new tube and centrifuged at 700× *g* for 4 min at RT. The pellet was resuspended in 200 µL of buffer solution. The suspension was loaded onto Abnova CytoQuest™ CR (Abnova Corp., Taipei City, Taiwan) as per the machine operation protocol. The CytoQuest machine induces a microfluidics flow over a chip (CytoChipNano-Abnova) that was previously coated with biotin-conjugated CSV antibody which binds the streptavidin on the chip. Upon completion of the isolation process, the cells on the CTC chip were subjected to live staining with fluorescein isothiocyanate (FITC)-conjugated CSV and PE-conjugated CD45 antibodies. All antibodies used for this process are part of the CytoQuest™ Universal Circulating Tumor Cell CSV CD45 Antibody Kit (KA4818 Abnova). Following staining, cells were fixed with 4% paraformaldehyde (PFA) solution in PBS (28906 ThermoFisher Scientific, Waltham, MA, USA) for 10 min at RT. After fixing, cells were blocked using buffer containing Fc receptor blocker and serum for 1 h at RT or overnight at 4 °C. Cells were then restained with CD45 followed by DAPI (D1306 ThermoFisher Scientific), a nuclear counterstain. All staining and fixing were carried out on the CTC chip using a fluid inlet for adding solutions and washing with PBS. The possibility of blood cells being incorrectly labeled as CTCs was accounted for by CD45 staining, to exclude lymphocytes. Of note, peritumoral CD45+ lymphocytes can also be seen surrounding CTCs and these cells are CSV negative [34].

**Figure 2:**
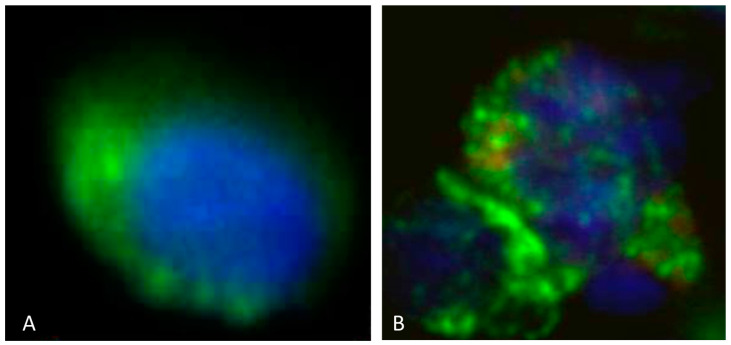
(**A**): CTC cell isolated from the peripheral blood from a patient with diffuse intrinsic pontine gliomas (DIPGs). The CTC displays rounded morphology and is CSV positive (displayed by green fluorescence) and H3K27M negative. The nucleus is seen in blue. (**B**): CTC cell isolated from the peripheral blood in a patient with DIPG. The CTC displays rounded morphology and is CSV positive (displayed by green fluorescence) and H3K27M positive (displayed by red fluorescence). The nucleus is seen in blue. Scale indicates 10 µm.

The criteria for CTC identification included CSV+ and CD45− staining, along with a minimum nuclear diameter of 10 µm as shown in Figure 2A. The minimum size aided in the size-based exclusion of PBMCs which are 7–10 µm in diameter and further support the identification of CTCs. As demonstrated in previous manual CSV+ CTC isolation studies, this assay is highly sensitive and specific [26,28,35].

### 2.3. Additional H3K27M Staining

Cells in the CytoChip were initially stained with only CSV as previously described followed by permeabilization with 0.25% NP40 in PBS supplemented with 1% FBS for 1 h. Following permeabilization, cells were stained with anti-H3K27M (0.05 µg/mL, Sigma St. Louis, MO, USA) for 1 h at RT and then stained with goat anti-human IgG (4 µg/mL) conjugated to PE for 1 h at RT before imaging. Figure 2B shows an isolated CTC from peripheral blood of a diffuse midline glioma patient with confirmed H3K27M tumor mutation which was stained for CSV and H3K27M by immunohistochemistry (IHC).

### 2.4. Automated Imaging and Enumeration

**Figure 3:**
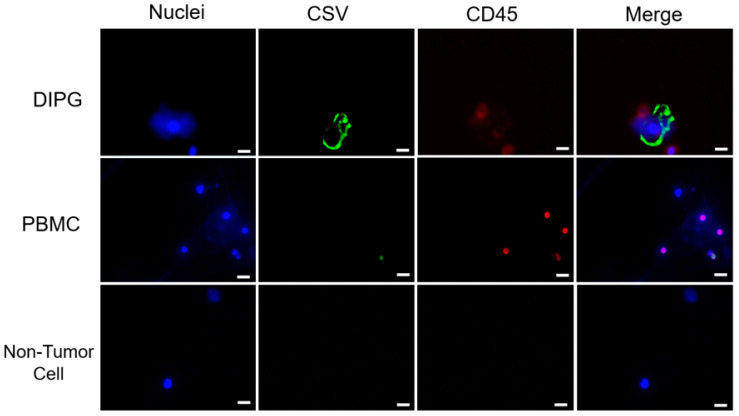
After single-cell dissociation from brain tumors, cells were stained with CD45 and CSV. Results of immunofluorescent staining of representative cells for CSV, nuclei and CD45 are shown. Immunofluorescent staining results for representative cells are shown. Scale indicates 10 µm.

CTCs were imaged using automated scanning on a Keyence BZ-X710 (Keyence Corporation, Itasca, IL, USA) immunofluorescence microscope. Imaged cells were manually identified and verified as CTCs as per mentioned criteria (CSV+, CD45−, ≥10 µm diameter). (Figure 3). Peritumoral CD45+ lymphocytes can also be seen with the DIPG CTC.

### 2.5. Cell Lines and Spiking Assay

**Figure 4:**
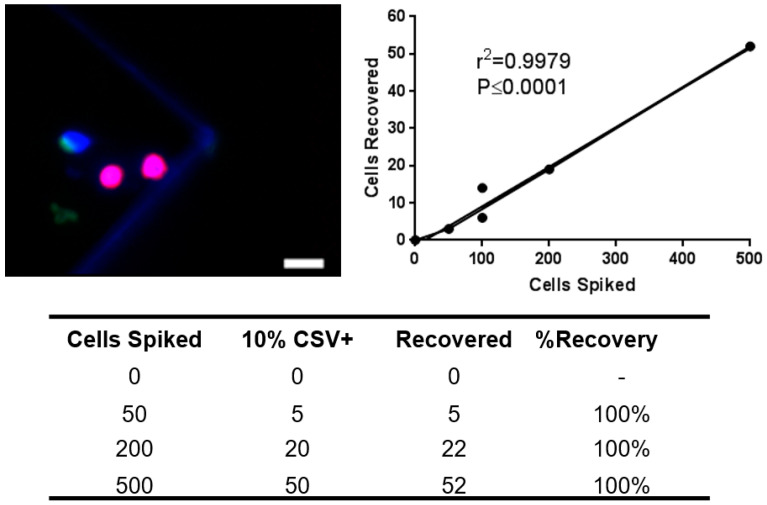
Cell line spiking and recovery. LN18 human glioblastoma cell line (10% CSV+) was spiked at 0, 50, 200 and 500 cells per 2 million healthy donor PBMCs per sample to demonstrate the recovery of glioblastoma cells during CTC isolation. Top left: Representative image of a recovered cells after spiking (blue = DAPI, green = CSV, red = CD45), scale bar indicates 10 µm. Top right: Linear regression analysis of recovery results. Bottom: Spiking assay recovery data.

LN18 cells were purchased from the American Type Culture Collection (ATCC) and used within 5–10 passages. Cells were grown and passed using the recommended media and protocol of the ATCC. LN18 cells used for the spiking assay were disassociated into a suspension using ethylenediaminetetraacetic acid (EDTA). Previous studies showed a highly CSV+ subpopulation of LN18 comprising 10% of all LN18 cells [28]. Based on these results, we spiked 0, 50, 200 and 500 cells into 2 million PBMCs from healthy donors per sample (Figure 4).

### 2.6. Statistical Analysis

Continuous variables were summarized using the median and range, and categorical variables were summarized using frequency and proportion. Data reported here are shown as mean ± standard error of the mean. A Wilcoxon rank-sum test and Fisher’s exact test were used to compare the continuous and categorical variables, respectively. The Spearman correlation coefficient was used to associate two continuous variables. A log-rank test and Cox proportional model were used to analyze overall survival. A two-sided *p*-value < 0.05 was considered significant. Multiple comparison was adjusted for in the analysis using FDR. The sensitivity and specificity of CTC counts for classifying patients with brain tumors versus healthy controls were assessed using receiver operating characteristic (ROC) analysis. The area under the ROC curve (AUC) was assessed and reported. The optimal cutoff points for CTC counts were obtained using the Youden index method. All analyses were performed in R 4.2.1. (R Development Core Team, Version 4.2.1) and/or GraphPad Prism software (https://www.graphpad.com/).

## 3. Results

### 3.1. Patient Demographics and Clinical Characteristics

Fifty-eight patients with CNS tumors were enrolled in the study and sixty-five peripheral blood samples were collected. Four samples were also collected from four healthy patients (two males and two females) with no history of malignancy and used as controls. Demographics for the 58 patients with CNS tumors is summarized in Table 1. Thirty-three patients were male (56.9%) and twenty-five patients (43.1%) were female. The median age at diagnosis was 10 (0.6–21) years. Of the 58 tumors, 13 (22.4%) were benign and 45 (77.6%) were malignant. Four (6.9%) patients had a cancer predisposition syndrome in this cohort, including Li–Fraumeni syndrome (*n* = 1), neurofibromatosis (NF) type 1 (*n* = 2) and NF2 (*n* = 1). Most patients either had high-grade glioma (*n* = 23; 39.6%: nineteen diffuse midline and four hemispheric), low-grade glioma (*n* = 16; 27.6%), medulloblastoma (*n* = 5; 8.6%) or others.

Seven patients (12.1%) had metastatic disease at the time of collection, one of which had extracranial metastatic disease. Samples were collected in 27 (46.6%) patients at diagnosis, 25 (43.1%) patients with progressive or refractory disease and in 6 (10.3%) patients who were in remission or off therapy. The median age at time of collection was 12.7 (2.1–23.5) years for patients with CNS tumors and 16 (12–18) years for healthy controls. For patients with CNS tumors, the median time of CTC sample collection after treatment (chemotherapy and/or radiation) for 65 samples was 41 (1–1446) days. Twenty patients (34.5%) were receiving steroid therapy at the time of initial sample collection. Forty-three patients underwent surgery prior to sample collection, of whom 22 (37.9%) had gross total resection, 12 (20.7%) subtotal resection and 9 (15.5%) had only a biopsy of the CNS tumor performed. Median time from surgery to CTC sample collection was 156 (10–4989) days.

### 3.2. CTCs Are Detectable in Pediatric CNS Tumor Patients

**Figure 5:**
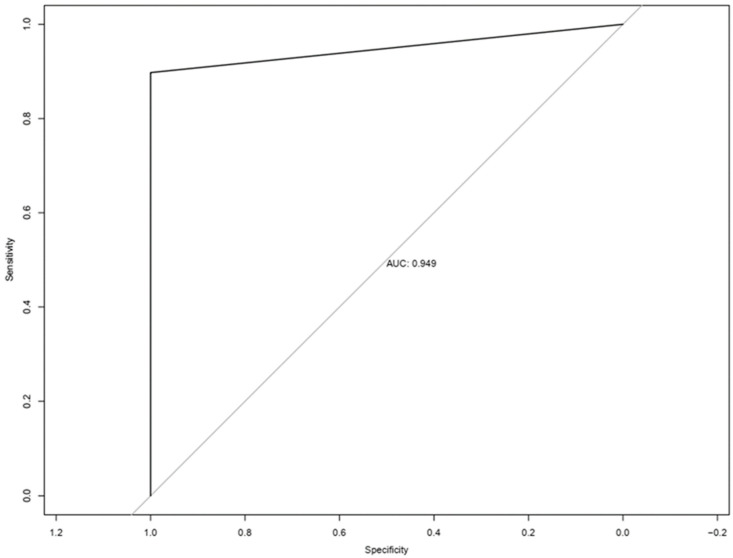
ROC curve generated based on CSV + CTCs of blood from patients with CNS tumors vs. healthy donors.

Of 58 patients, CTCs were detected in 50 (86.2%) patients. Our results did not demonstrate any significant difference in CTC positivity between genders, age groups, benign vs. malignant, metastatic status or disease status at time of collection. The incidence of CTC detection did not correlate with use of systemic steroids, chemotherapy or radiation received, disease status, presence of metastatic disease or peripheral blood cell count at time of sample collection. The median number of CTCs detected was 2 (0–13). Using this information, a ROC curve was constructed (Figure 5). The AUC of the ROC curve was 0.949. As the Youden J value was one or more CTCs detected, this level was used as the cutoff for CTC positivity. Using this cutoff value, the sensitivity and specificity of this test for discrimination of patients from healthy controls were 86.2% and 100%, respectively, with a positive predictive value of 100% and negative predictive value of 33.3%.

### 3.3. Midline Glioma Cohort

For the 19 patients with midline glioma, there were 6 males and 13 females, diagnosed at a median age of 7 (2–19) years. Ten (52.6%) patients were positive for H3K27M mutation and mutation status was unknown for nine (47.4%) of patients. Of these 19 patients, peripheral blood samples were collected from seven (36.8%) patients at diagnosis and nine (47.4%) upon disease progression and three (15.8%) with stable disease. CTCs were detected in 15 (78.9%) patients with midline gliomas and the median number of CTCs detected was 2 (0–8).

We additionally stained a portion of the DIPG and midline glioma samples for H3K27M, a biomarker that has been associated with worse prognosis in these patients. This also further confirmed that the CSV+ cells larger than 10 µM were in fact CTCs. We found that of the eight DIPG and midline glioma samples stained, H3K27M+ CTCs could be detected in six samples (75.0%). A representative image can be seen in Figure 2B. Patients with H3K27M+ CTCs had a lower median overall survival of 271 (166–421) days vs. patients with H3K27M- CTCs of 309.5 (287–332) days but this was not statistically significant (*p*-value = 0.6), possibly due to the small sample size (Table 2).

## 4. Discussion

This study is the first to demonstrate the detection of CTCs using CSV as a biomarker in pediatric CNS tumors, including GBM and non-GBM tumors such as embryonal tumors such as medulloblastoma and atypical teratoid rhabdoid tumors (ATRTs). Prior studies examined the isolation and capture of CTCs in the blood of CNS tumor patients using size- or affinity-based methods. These include telomerase promoter-based assays or the use of a variety of tumor markers including oncofetal chondroitin sulfate, a GBM CTC antibody cocktail, glial fibrillary acidic protein (GFAP) and polyploidy detection by FISH with variable reported CTC isolation rates of 20.6–77% [31,32,35,36,37,38]. Multiple size-based detection methods have also been explored using microfluidic size exclusion devices or stationary size exclusion-based cell isolators, also with variable detection rates, with one size-based platform reporting a 39% (13 of 33) detection rate of CTCs in patients with GBM [33,37]. Other liquid biopsy-based approaches focused on CNS tumors have analyzed cfDNA and miRNA in the CSF.

Unlike previously published studies, our approach relies on CSV, a mesenchymal protein that is mislocalized on the surface of tumor cells, while remaining cytosolic in non-tumor mesenchymal cells. CSV is a universal CTC capture antibody that our lab has previously validated in both epithelial and mesenchymal tumor-bearing patients [28,35,39]. This tool has been successfully documented for CTC capture in breast, prostate, lung, sarcoma and neuroblastoma cancers [26,35,40]. We have previously published CTC studies using manual isolation following the same principles of CSV-based CTC identification and isolation [26,34,36]. Here, we present an automation of our approach using the Abnova CytoQuest™ CR platform. In addition to decreased processing time, we have observed increased sensitivity in spiking assays. Whereas we previously recorded 80–85% sensitivity of detection, we are now consistently observing 95% or greater spiked cell detection rates, though with increased PBMC background. This streamlined and efficient process can provide faster sample processing and data analysis. Larger studies, however, are needed to further confirm the absence of CTCs in healthy controls and exclude the possibility of false positive results.

Additionally, using our CSV-based detection and capture method, our results show a CTC detection rate of 86.2% which is higher than previously reported using different methods as outlined above. Similarly, in this study the rate of CTC detection in patients with midline gliomas was much higher at 78.9% vs. 20.6–39% as previously reported in adult patients with GBM. Again, this is likely due to differences in methodology as GBMs are highly heterogenous tumors and these studies relied on the identification of a variety of proteins whose expression may be variable, including glial fibrillary acidic protein (GFAP) in one study and SRY-Box transcription factor 2 (SOX2), tubulin beta-3, epidermal growth factor receptor (EGFR), A2B5 (a ganglioside marker) and c-MET (a tyrosine-protein kinase) in others [32,33]. 

Of note, although the CTC detection rate in patients with midline gliomas was higher in our study than previously reported, overall, it was still lower in patients with midline gliomas (15/19: 78.9%) compared to other tumors (35/39: 90%). Historically, these tumors have a very low rate of neural metastases of only 10% and very rarely disseminate extraneurally. Additionally, the relatively lower incidence of CTCs in this population compared to other CNS tumors may be due to the origin of the primary tumor itself, which is situated in the glial cells of the brainstem, as well as the heterogenous nature of the blood–brain barrier and its increased permeability in other tumors versus patients with diffuse midline gliomas [41]. Furthermore, 34.5% of all patients in this study were receiving corticosteroids at the time of collection which is known to have anti-inflammatory effects and decrease the permeability of the blood–brain barrier, potentially decreasing the migration of CTCs into the peripheral blood [42]. Conversely, patients undergoing radiation may have increased disruption of the blood–brain barrier, resulting in greater CTC dissemination.

Interestingly, this is the first study to report the isolation of CTCs in benign tumors such as low-grade gliomas. While CTCs are associated with increased metastatic capacity, only a small proportion of CTCs persist in the peripheral circulation and eventually initiate metastases [43,44]. Larger studies are needed to further elucidate this finding and understand the complex interactions between these tumor cells and the microenvironment leading to metastases. Serial CTC measurements would also be beneficial in understanding the duration of persistence of these cells, how they vary between tumor types and their prognostic impact.

As previously mentioned, there is currently no validated method of detecting minimal residual disease in pediatric patients with CNS tumors. Instead, for disease surveillance, these patients are subjected to serial neuroimaging at regular intervals and/or CSF cytology evaluation which pose several challenges. In addition to resulting in the need for repeated sedation in small children and increased health care costs, standard MRI provides limited insight into the heterogenous nature of the tumor and imaging findings lag behind tumor progression in real time [45]. Furthermore, up to 46% of patients may be asymptomatic at the time of disease relapse detection on imaging, highlighting the dire need for more sensitive methods of earlier disease detection [46]. Given the clinical need, there has been concerted effort put into discovering minimally invasive reliable liquid biomarkers for patients with CNS tumors. While larger studies are needed to validate the findings in this study, the detection of CTCs in patients with CNS tumors can potentially be used to confirm the diagnosis of inoperable, difficult to biopsy tumors such as brainstem gliomas and optic gliomas as well as serially monitor for residual disease prior to detectable radiographic changes. CTCs may also be beneficial in helping to distinguish pseudoprogression due to treatment from true progression which is often indistinguishable on radiographic imaging. Finally, CTCs can provide molecular insight into tumor biology and can help direct targetable treatment such as H3K27M in DIPG and BRAF in patients with low-grade gliomas.

## 5. Conclusions

To gain a better understanding of disseminated CNS tumor cells and to explore the possibility of the clinical relevance of captured CTCs, we initiated this study using CSV as a biomarker in pediatric patients. Our results support previous evidence of CTCs in adult CNS tumor patients but have also shown, for the first time, that CTCs can be isolated from pediatric patients with CNS tumors.

In summary, this study demonstrates the utility of using CSV in pediatric and AYA patients as a biomarker to detect CTCs. This finding may allow the early identification of treatment failure or relapse of patients who may benefit from modified therapies. This also opens the door for molecular characterization of the isolated CTCs with potential detection of targetable mutations or markers. This is of utmost importance particularly in patients with inoperable CNS tumors or in those where a biopsy is not feasible and may result in increased morbidity and mortality such as DIPG or optic gliomas. Additional studies are needed to further validate these results and define their potential role in longitudinal disease monitoring, providing tumor molecular insight and developing risk-adapted, personalized treatment strategies.

## Figures and Tables

**Table 1:** Summary of Patient Demographics with Central Nervous System Tumors.

Characteristic		No of Patients (%) (*n* = 58)	No of CTC Samples (%) (*n* = 65)
Median age at diagnosis (years)		10 (0.6–21)	
Gender	Male	33 (56.9%)	
	Female	25 (43.1%)	
Performance status (Lansky/Karnofsky)	90–100	35 (60.4%)	
	60–80	19 (32.7%)	
	30–50	4 (6.9%)	
	0–20	0	
Cancer predisposition	Yes	4 (6.9%)	
	No	54 (93.1%)	
Tumor	Benign	13 (22.4%)	
	Malignant	45 (77.6%)	
Location	Hemisphere	15 (25.9%)	
	Optic pathway	4 (6.9%)	
	Thalamus	1 (1.7%)	
	Brainstem	18 (31.0%)	
	Cerebellum	7 (12.1%)	
	Pineal gland	3 (5.2%)	
	Suprasellar	5 (8.6%)	
	Ventricular	3 (5.2%)	
	Spine	1 (1.7%)	
	Other	1 (1.7%)	
Histology	Midline high-grade glioma	19 (32.7%)	
	Hemispheric high-grade glioma	4 (6.9%)	
	Low-grade glioma	16 (27.6%)	
	Medulloblastoma	5 (8.6%)	
	ATRT	1 (1.7%)	
	PNET	2 (3.5%)	
	Craniopharyngioma	3 (5.2%)	
	GCT	2 (3.5%)	
	Ependymoma	1 (1.7%)	
	Other	5 (8.6%)	
Metastases	Yes	7 (12.1%)	
	No	51 (87.9%)	
Surgery	GTR	22 (37.9%)	
	STR	12 (20.7%)	
	Biopsy	9 (15.5%)	
	None	15 (25.9%)	
Disease status	New diagnosis	27 (46.6%)	27 (41.5%)
	Refractory/progressive disease	25 (43.1%)	32 (49.3%)
	Survivor/remission	6 (10.3%)	6 (9.2%)
Median time of collection of CTC sample after treatment (days)			41 (1–1446)
Median platelet count at time of CTC collection (×10^3^/µL)			266 (16–568)
Median APC at time of CTC collection (×10^3^/µL)			3.72 (0.16–14.58)
Median ANC at time of CTC collection (×10^3^/µL)			3.28 (0.11–13.0)
Patient receiving steroids at time of collection	Yes	20 (34.5%)	23 (35.4%)
	No	38 (65.5%)	42 (64.6%)
CTCs detected	Yes	50 (86.2%)	55 (84.6%)
	No	8 (13.8%)	10 (15.4%)
No of CTCs		2 (0–13)	2 (0–13)
No of CTCs/mL		0.5 (0–2.5)	0.5 (0–2.5)

ATRT: Atypical Teratoid Rhabdoid Tumor; PNET: Primitive Neuroectodermal Tumor; GCT: Germ Cell Tumor; GTR: Gross Total Resection; STR: Subtotal Resection; CTC: Circulating Tumor Cell, µL: Microliter; APC: Absolute Phagocyte Count, ANC: Absolute Neutrophil Count; mL: Milliliter.

**Table 2:** Characteristics of Patients with diffuse midline glioma.

Demographic		No of Patients (%)
Total		19
Median age at diagnosis (years)		7 (2–19)
Gender	Male	6 (31.6%)
	Female	13 (68.4%)
HK327M status	Positive	10 (52.6%)
	Negative	9 (47.4%)
Disease status	New diagnosis	7 (36.8%)
	Refractory/progressive disease	9 (47.4%)
	Stable disease	3 (15.8%)
CTCs detected	Yes	15 (78.9%)
	No	4 (21.1%)
No of CTCs		2 (0–8)
HK327M CTCs detected (*n* = 8)	Yes	6 (75.0%)
	No	2 (25.0%)
Overall survival (days)	H3K27M-positive CSV+ CTCs	271 (166–421)
	H3K27M-negative CSV+ CTCs	309.5 (287–332)

## Data Availability

The data that support the findings of this study are available from the corresponding author upon reasonable request.
